# A No-Biopsy Approach for the Diagnosis of Celiac Disease in Adults: Can It Be Real?

**DOI:** 10.7759/cureus.26521

**Published:** 2022-07-03

**Authors:** Ahmed Ramiz Baykan, Serkan Cerrah, Sedat Ciftel, Mete Koray Vural, Elmas Kasap

**Affiliations:** 1 Gastroenterology and Hepatology, Erzurum Regional Training and Research Hospital, Erzurum, TUR; 2 Medical Microbiology, Erzurum Regional Training and Research Hospital, Erzurum, TUR; 3 Gastroenterology and Hepatology, Manisa Celal Bayar University, Manisa, TUR

**Keywords:** villous atrophy, serological tests in celiac disease, no-biopsy approach, tissue transglutaminase antibody, celiac disease

## Abstract

Background and objective

Pediatric guidelines on the diagnosis of celiac disease (CD) have reported that the positivity of anti-endomysium antibodies in the presence of anti-transglutaminase antibodies (TGA) 10 times higher than normal is sufficient for the diagnosis. In this study, we aimed to evaluate whether this diagnostic process for children can also be applied to adult patients.

Materials and methods

We retrospectively examined patients aged >18 years who were diagnosed with CD. The results of serological tests and endoscopic biopsy were evaluated. Patients with more than one month of duration between celiac serology and endoscopy, those diagnosed with CD before admission, those on a gluten-free diet, and those with selective IgA deficiency were excluded from the study.

Results

A total of 269 patients were included in the study. TGA value was significantly higher in patients with villous atrophy (p<0.001) and positively correlated with mucosal damage (r=0.60, p<0.01). Considering the cut-off value of 100 U/mL (>10 ULN) for the TGA antibodies, in line with the criteria regulated by the European Society for Paediatric Gastroenterology, Hepatology, and Nutrition (ESPGHAN) for the diagnosis of CD, the sensitivity was 71.64%, the specificity was 100%, and the positive predictive value (PPV) was 100%. When the cut-off value was taken as 29.42 U/mL, the sensitivity was 100% and the specificity was 99.5%. For a TGA cut-off value of 52.7 U/mL (5.27 ULN), which determines the presence of partial or complete villous atrophy in the evaluation made considering mucosal damage, the sensitivity was 90%, the specificity was 100%, and the PPV was 100%.

Conclusion

Based on our findings, TGA titers were highly effective in demonstrating CD-related mucosal damage. This study endorses a biopsy-free strategy in adult patients in line with the ESPGHAN criteria. Local validation of test-specific thresholds will ensure that this approach has a significant impact on adult patients.

## Introduction

Over the past 70 years, the diagnosis of celiac disease (CD) has evolved from symptom-based inferences to the use of complex serological and histological methods. The detection of duodenal mucosal damage, development of biopsy techniques, and sampling have been a cornerstone in the diagnosis of CD [1,2]. Although the sensitivity and specificity were low in the 1980s, serological tests, such as endomysial antibodies, were used as the first screening tests [3]. Another important milestone in the diagnosis and etiopathogenesis of CD was the discovery of tissue transglutaminase 2 in 1997 [4]. In fact, tissue transglutaminase 2, which is a gut enzyme, functions not only as a target autoantigen but also as a deamidation enzyme that can enhance the immunostimulatory effect of gluten [5]. As a result, gliadin interacts with human leukocyte antigen (HLA)-DQ2 (or HLA-DQ8) on the surface of antigen-presenting cells as it is deaminated by tissue transglutaminase 2, and its presentation to CD4 T cells via the T cell receptor results in cytokine release and tissue damage. Malabsorption is the result of mucosal damage caused by humoral and cell-mediated autoimmunity [6].

The clinical features of the disease range from typical gastrointestinal findings to rather vague extra-intestinal findings. Therefore, most of the patients remain undiagnosed [7]. Today, the diagnosis of CD in adults is traditionally made based on serological tests and the detection of characteristic histopathological findings in endoscopic biopsy specimens. The European Society for Paediatric Gastroenterology, Hepatology, and Nutrition (ESPGHAN) stated in 2012 that the diagnosis of CD in children can be made based on the presence of appropriate symptoms, such as an IgA tissue transglutaminase antibody (TGA) level that is at least 10 times higher than normal, positive anti-endomysium (EMA) antibodies, and an appropriate HLA genotype [8]. Although this is a huge leap forward, it has paradoxically generated new controversy, as these guidelines are not currently adopted in all countries and are not intended for adults. In 2020, the guidelines were revised, and the criteria regarding the presence of appropriate symptoms and the necessity of the HLA test in the diagnosis were removed, meaning that only TGA levels at least 10 times higher than normal and the presence of positive EMA antibodies were deemed sufficient for the diagnosis [9]. This result diminishes the importance of endoscopy in the diagnosis of CD. In the current study, we aimed to evaluate the endoscopic biopsy and serological results associated with CD diagnosis for the first time, and evaluate whether this diagnostic process for children can also be applied to adult patients.

## Materials and methods

Patients

This research was designed as a retrospective cohort study. Patients who presented to the Gastroenterology Clinic of the Erzurum Regional Training and Research Hospital between January 1, 2018, and January 1, 2019, who underwent a serological test for CD and were also sampled for CD on endoscopy were included. Patients younger than 18 years of age, those with more than one month of duration between celiac serology and endoscopy, those with a previous diagnosis of CD, those on a gluten-free diet, and those with selective IgA deficiency were excluded from the study. The study, in accordance with the Declaration of Helsinki, was granted approval by the Erzurum Training and Research Hospital Ethics Committee (ethics committee approval number: 2021/23-293).

Histology and serology

TGA, EMA, and antigliadin antibodies were sampled from patients who fulfilled the appropriate criteria. Serology was studied using the ELISA kit (Orgentec, Mainz, Germany) and an Alisei QS (SEAC Group, Italy) device. The results were evaluated in U/mL and the cut-off value for the TGA was taken as 10 U/mL.

The endoscopic evaluation was performed by three gastroenterologists experienced in their field. The biopsy was performed from at least four different locations in the duodenum, including the bulb. In the pathological evaluation, the samples were categorized according to the Marsh Classification based on the mucosal damage. The patients were classified into three groups: Marsh 0 (normal mucosa), Marsh 1 and 2, and Marsh 3a-3c (partial or total villous atrophy).

Statistical analysis

SPSS Statistics for Windows 17.0 (IBM, Armonk, NY) was used for statistical analysis. Graphpad Prism 9.3.1 (GraphPad Software, San Diego, CA) was used for generating the graphs. Numerical variables with normal distribution were presented as the mean ± SD, and those that did not show a normal distribution were presented as the median (minimum-maximum). Categorical variables were presented as numbers and percentages. The Mann-Whitney U and Kruskal-Wallis H tests were used for the intergroup comparisons of the non-normally distributed numerical variables. Categorical variables were compared using the χ2 and Fisher's exact χ2 tests. Spearman correlation analysis was used for determining the relationship between the TGA and mucosal injury. ROC analysis was used to determine the cut-off value of the optimum TGA for the diagnosis of CD and predict the degree of duodenal damage.

## Results

Study participants

The study involved a total of 269 patients, comprising 165 (61.3%) females and 104 (38.7%) males. The mean age was 31.79 ± 1.01 years among males and 31.88 ± 0.84 years among females (p=0.94).

Status of the patients based on their serological diagnosis

The TGA value of 195 patients was below 10 U/mL. Although villous atrophy was not observed in any of these patients, an increase in the intraepithelial lymphocyte (IEL) or crypt hyperplasia (Marsh 1 or 2) was detected in 57 patients. The TGA value was >10 U/mL in 74 (27.5%) patients. Moreover, 64 (86.5%) of these patients had partial or total villous atrophy (Marsh 3a-3c). This resulted in crypt hyperplasia (Marsh 2) in seven of the remaining 10 patients (Table 1).

EMA antibodies were found to be present in 81 (30%) patients after the patients with normal duodenal mucosa were excluded. Of these, 17 were classified as Marsh 1 or 2 with mild mucosal damage, and the remaining 64 patients were classified as Marsh 3 (Table 1).

**Table 1:** Distribution of the patients according to the serological and histological data

	IgA anti-transglutaminase			IgA anti-endomysial antibody
	<10 U/mL, n (%)	10–100 U/mL, n (%)	>100 U/mL, n (%)	Number and rate of positive results
Marsh 0	138 (51.3%)	3 (1.1%)	0	21/141 (14.9%)
Marsh 1	50 (18.6%)	1 (0.4%)	0	12/51 (23,5%)
Marsh 2	7 (2.6%)	6 (2.2%)	0	5/13 (38.5%)
Marsh 3a	0	12 (4.5%)	15 (5.6%)	27/27 (100%)
Marsh 3b	0	4 (1.5%)	18 (6.7%)	22/22 (100%)
Marsh 3c	0	0	15 (5.6%)	15/15 (100%)

Anti-gliadin IgA antibodies were present in nine (6.7%) patients with normal mucosa, six (10%) patients with Marsh classification 1-2, and 45 (73.7%) patients with Marsh classification 3a-3c.

Relationship between the TGA level and mucosal damage

**Figure 1:**
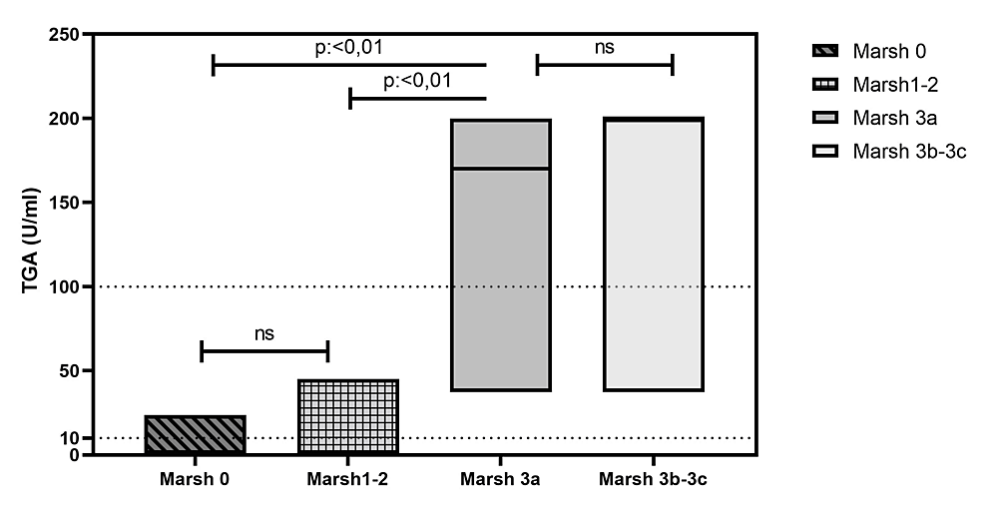
TGA titers and Marsh grades in patients P<0.05 is considered significant for statistical analyses ns: nonsignificant; TGA: anti-transglutaminase antibody

When the TGA values were compared with the mucosal damage, there were no significant differences between the TGA levels in the presence of normal mucosa (Marsh 0) or increased lymphocyte or crypt hyperplasia (Marsh 1-2). In the presence of partial or total mucosal atrophy (Marsh 3a-3c), the TGA values were significantly higher when compared to the other groups. There were no significant differences in the TGA levels between patients with Marsh classification 3a-3c (Figure 1).

When the correlation between the TGA levels and mucosal damage was evaluated, it was seen that the mucosal damage and TGA levels were positively correlated in the Spearman analysis (r=0.60, p<0.01).

Sensitivity and specificity of the TGA antibodies at different cut-offs

Considering the cut-off value of 100 U/mL (>10 ULN) for the TGA antibodies, in line with the criteria regulated by ESPGHAN for the diagnosis of CD, the sensitivity was 71.64%, the specificity was 100%, and the positive predictive value (PPV) was 100%. When the cut-off value was taken as 29.42 U/mL, the sensitivity was 100% and the specificity was 99.5% (Table 2). For a TGA cut-off value of 52.7 U/mL (5.27 ULN), which determines the presence of partial or complete villous atrophy in the evaluation made considering mucosal damage, the sensitivity was 90%, the specificity was 100%, and the PPV was 100% (Table 3). The different cut-off values for the TGA in the diagnosis of CD are given in Table 4.

**Table 2:** Sensitivities and specificities of different cut-off values for TGA in detecting celiac disease TGA: anti-transglutaminase antibody; PPV: positive predictive value; NPV: negative predictive value

	TGA cut-off value		
	>10 U/mL	>29.42 U/mL	>100 U/mL
Sensitivity, %, value (95% CI)	100 (94.64–100)	100 (94.64–100)	71.64 (59.31–81.99)
Specificity, %, value (95% CI)	96.53 (92.99–98.6)	99.50 (97.27–99.99)	100 (98.19–100)
PPV, %, value (95% CI)	90.54 (82.21–95.2)	98.53 (90.46–99.79)	100
NPV, %, value (95% CI)	100	100	91.40 (87.90–93.96)

**Table 3:** Diagnostic values of the serum transglutaminase antibodies for different mucosal damages in celiac disease *P<0.05 is considered significant for statistical analyses TGA: anti-transglutaminase antibody; AUC; area under the curve

	TGA cut-off value (U/mL)	Sensitivity	Specificity	AUC	P-value
Marsh 1	2.00	71%	58%	0.74	<0.01*
Marsh 2	4.3	94%	90%	0.96	<0.01*
Marsh 3	52.7	90%	100%	0.99	<0.01*

**Table 4:** TGA values in the diagnosis of celiac disease at different cut-off levels. TGA: anti-transglutaminase antibody; PPV: positive predictive value

TGA cut-off value	Sensitivity, %		Specificity, %		PPV, %	
	Value	95% CI	Value	95% CI	Value	95% CI
21.74 U/mL	100%	94.64–100	99.0%	96.47–99.88	97.1%	89.40–99.25
29.42 U/mL	100%	94.64–100	99.5%	97.27–99.99	98.5%	90.46–99.79
39.69 U/mL	95.5%	87.47–99.07	100%	98.19–100	100%	
41.31 U/mL	92.5%	83.44–97.53	100%	98.19–100	100%	
TGA >10 and villous atrophy						
29.42 U/mL	100%	94.40–100	98%	95.08–99.47	94.1%	85.84–97.69
36.1 U/mL	100%	94.40–100	98.5%	95.78–99.7	95.5%	87.40–98.50
39.69 U/mL	96.9%	89.16–99.62	99%	96.52–99.88	96.8%	88.64–99.19
41.31 U/mL	93.8%	84.76–98.27	99%	96.52–99.88	96.7%	88.29–99.17
52.7 U/mL	90.6%	80.70–96.48	100%	98.22–100	100%	

## Discussion

In this study, we evaluated the applicability of this diagnostic method in adult patients, in line with the recommendations of ESPGHAN, based on the recommendation of at least a 10-fold increase in IgA-TGA antibodies, positive EMA being sufficient for the diagnosis of CD, and the application of endoscopy for patients who do not meet the diagnostic criteria. It was observed that when the IgA-TGA antibodies were >10 ULN, and in the case of positive EMA, the sensitivity for the diagnosis of CD was 71.64%, the specificity was 100%, and the PPV was 100%. We also found that sensitivity was 100%, specificity was 99.5%, and PPV was 98.5% when the cut-off value was taken as 29.42 U/mL.

Until 2012, the histological evidence of villous atrophy in small bowel biopsies was mandatory for the diagnosis of CD. In the last 10 years, a strong correlation was found between TGA titer levels and the severity of mucosal lesions, while the clarity of the histopathology was questioned [10]. In their observational study, Tortora et al. [11] estimated that the best threshold value for Marsh classification 2 was 6.4 ULN (PPV and specificity: 100%, sensitivity: 70%) and 8.9 ULN (sensitivity: 69%, specificity: 100%) for Marsh classification 3. Zanini et al. [12], in their study involving 945 patients, reported that the specificity was 100% for villous atrophy if the TGA value was 5 ULN. Similarly, in the current study, it was observed that the TGA level was correlated with mucosal injury (r=0.60, p<0.01), and the sensitivity was 90%, the specificity was 100%, and the PPV was 100% when the cut-off value for Marsh classification 3 was taken as >52.7 U/mL (5.27 ULN).

Cut-off values other than the ESPGHAN TGA >10 ULN recommendations have been suggested for the diagnosis of CD in various studies. Losurdo et al. [13] reported that a TGA level >6.2 ULN in adults represented the best diagnostic value (sensitivity: 57.14%, specificity: 65.59%, PPV: 82.4%). They explained that the diagnostic value was lower than in other studies due to the different population cohorts and controls in their study. In their analysis of 270 adult patients, Holmes et al. [14] determined that the PPV was 100% for CD if the TGA was >45 U/mL (8 ULN). The different test kits cause a problem regarding standardization in the diagnosis [15]. In their multicenter study including seven Mediterranean countries and 1,974 patients and using eight different kits, Smarrazzo et al. [16] found that a TGA level >1.25 ULN was quite reliable for measurements, and detected the PPV to be 96.1% in cases where the TGA level was >10 ULN. In another Italian study [17] involving 234 patients, the PPV was determined to be 97.66% in the diagnosis of TGA based on the ESPGHAN criteria (>10 ULN). Di Tola et al. [18] found the best cut-off value with a TGA level >3.6 ULN, where the PPV was detected as 97.2%. In the current study, it was determined that the best cut-off value was 29.42 U/mL (>2.94 ULN) (sensitivity: 100%, specificity: 99.5%, and PPV: 98.53%). It was observed that the sensitivity decreased by 71.64% and the specificity and PPV values were 100% when based on a TGA level >10 ULN.

Although excellent predictive values have been reported in similar studies, including the current study, it seems that duodenal biopsy will remain a part of the diagnosis for a while. In fact, the British Society of Gastroenterology has stated that the biopsy-free approach in adults is premature due to the incompatibility between kits [19]. Finally, in the guidebook proposed by the European Society for the Study of Coeliac Disease (ESsCD) [20] in 2019, it was stated that the subject is under investigation and more data are needed before a recommendation can be made. Regional differences with respect to TGA levels also seem to be an important issue. In publications from North America, it was reported that TGA tests have poor accuracy, although the reasons for this were unclear [15].

The biggest handicap of the biopsy-based diagnostic approach is the difficulties experienced in the diagnosis due to patchy involvement and indistinct histopathological changes due to early disease. False-negative results are more common than false positives in the diagnosis. Misinterpretations vary between 10% and 20% due to various reasons [21]. The villous height-to-crypt depth ratio (Vh/CrD), the densities of CD3+, ɤδ+, and villous type IELs are promising developments in histological diagnosis [22].

This study has some other limitations in addition to its retrospective nature. In some studies, it is stated that different TGA commercial kits have a high variability rate [15,16]. We did not use different commercial TGA kits in our study, and we consider this to be the most important limitation of our study. In this regard, there is a need for new studies to be conducted.

## Conclusions

The findings of this study showed that TGA titers are highly effective in identifying adults with CD-related intestinal changes. This study endorses a biopsy-free strategy in adult patients in line with the ESPGHAN criteria. This approach has implications for reducing the cost, risk, and caseload associated with diagnostic endoscopy in adult CD. However, local validation of the test-specific thresholds will allow this approach to have a major impact on adult patients.
